# Remote cerebellar hemorrhage and intracranial hypotension syndrome following pituitary surgery

**DOI:** 10.1590/0100-3984.2015.0089

**Published:** 2016

**Authors:** Luiz de Abreu Junior, Henrique T. Martucci, Paulo Tarso Reck de Mendonça, Gustavo Garcia Marques, Célia Rodrigues

**Affiliations:** 1 Universidade São Camilo, São Paulo, SP, Brazil.; 2 Clínica São Camilo, Sinop, MT, Brazil.; 3 Instituto Neurocirúrgico de Sinop, Sinop, MT, Brazil.

*Dear Editor*,

A 62-year-old male presented with a several-month history of headache and alteration in his visual field. The diagnostic evaluation revealed a suprasellar mass that was causing hydrocephalus by extrinsic compression. We opted for ventricular shunt placement and subsequent surgical excision of the mass. The surgical site was accessed through left frontal craniotomy. Histological analysis of the resected mass revealed that it was a craniopharyngioma.

**Figure 1:**
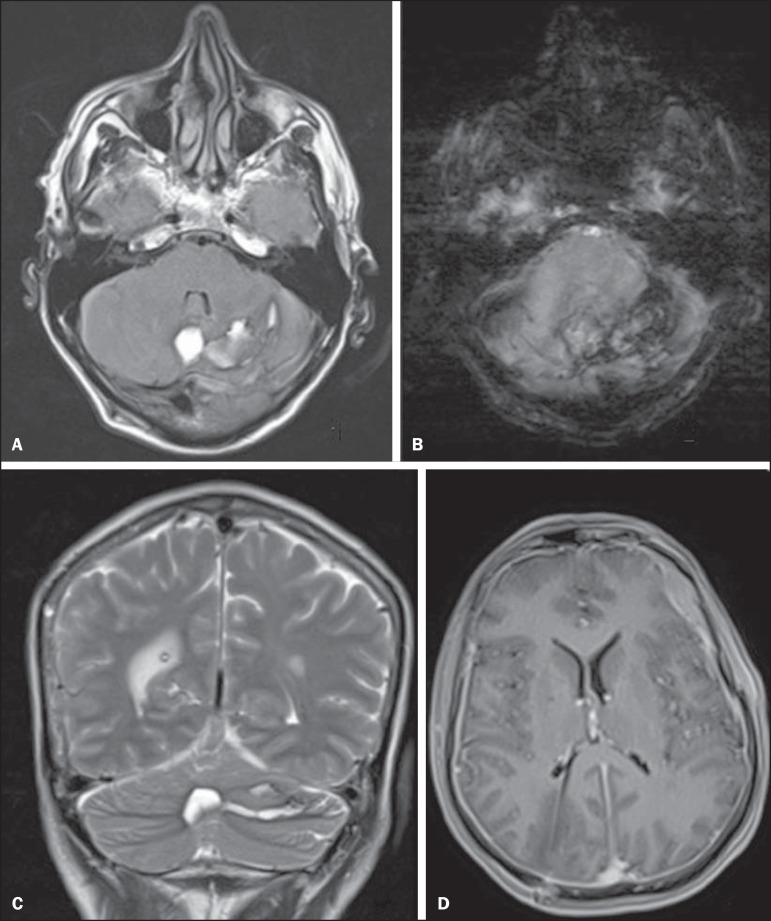
MRI scans of the cranium. **A:** FLAIR sequence in the axial plane, showing foci of hyperintense signals in the vermis and the left cerebellar hemisphere, which is consistent with cerebellar hemorrhage. **B:** SWI sequence in the axial plane, showing marked hypointensity indicative of hemoglobin degradation products (e.g., hemosiderin), with the zebra sign (pattern of distribution among the cerebellar folia). **C:** T2-weighted TSE sequence in the coronal plane, confirming cerebellar hemorrhage, in the different phases of hemoglobin degradation, with alternating areas of hyperintense and hypointense signals (the zebra sign). **D:** T1-weighted SE sequence in the axial plane after intravenous injection of paramagnetic contrast, showing the terminus of the ventricular shunt catheter at the trigone of the right lateral ventricle. Note the small size of the lateral ventricles, together with the marked dural thickening and significant dural impregnation, as well as the hematic left frontal subdural fluid collection. This combination of findings suggests a loss of cerebrospinal fluid (intracranial hypotension syndrome).

In the postoperative period, the patient evolved to significant worsening of the headache, and a magnetic resonance imaging scan was requested in order to evaluate the condition. The magnetic resonance imaging showed cerebellar hemorrhage affecting the vermis and the left cerebellar hemisphere (Figure 1A), with standard distribution of blood among the cerebellar folia (Figures 1B and 1C), indicative of remote cerebellar hemorrhage. There were also signs suggestive of a loss of cerebrospinal fluid (intracranial hypotension syndrome), such signs including a decrease in the size of the ventricles, dural thickening, diffuse dural enhancement, and hematic left frontal subdural fluid collection (Figure 1D).

Remote cerebellar hemorrhage has been defined as bleeding within the cerebellar parenchyma, a rare complication that can occur after neurosurgical intervention. The entity was first described in the 1970s, by Yasargil et al.^(1)^. The reported incidence of remote cerebellar hemorrhage after supratentorial interventions ranges from 0.08% to 0.6%^(2)^. However, it has been reported to occur after various other surgical procedures involving the cranium or spinal cord^(2-7)^.

Several hypotheses have been suggested to explain the appearance of bleeding in the cerebellum away from the primary (supratentorial or spinal) surgical site. One such hypothesis is that resection of a supratentorial lesion creates a pressure gradient, resulting in suction on the cerebellar veins, particularly in the upper portion of the vermis^(8)^. However, there is another hypothesis that might explain the two findings in the case reported here. That hypothesis is based on the supposition that opening the cisterns or the ventricular system promotes intracranial hypotension, triggering the process that culminates in the distension and rupture of cerebellar veins, resulting in cerebellar hemorrhage ^(9)^.

Various neurosurgical procedures have been associated with the occurrence of remote cerebellar hemorrhage, including the clipping of aneurysms (ruptured or otherwise), tumor resection, drainage of parenchymal or extra-axial hematomas, and spinal surgery^(2-7,9)^. In imaging examinations, remote cerebellar hemorrhage has a characteristic presentation, with a tendency for the blood to be distributed among the cerebellar folia with a curvilinear configuration. This aspect results in the pattern known as the zebra sign^(8)^.

The symptoms of intracranial hypotension syndrome include headache that is orthostatic in presentation, tending to improve in the recumbent position. In imaging studies of the brains of patients with intracranial hypotension^(10)^, findings include dural thickening and diffuse dural enhancement; engorgement and dilatation of venous structures; subdural fluid collections; downward displacement of the midbrain; and herniation of the cerebellar tonsils.

The case presented here demonstrates a chain of events that could have collectively resulted in the two central nervous system complications observed. The supratentorial surgical manipulation and the placement of the ventricular shunt could have caused intracranial hypotension, resulting in the traction, distension, and consequent rupture of cerebellar veins, as well as hemorrhage in the cerebellar parenchyma.

Radiologist knowledge of these entities is relevant, because their proper, early characterization can promote interventions aimed at their correction and at alleviating the associated symptoms.
